# Microarray-based detection and expression analysis of drug resistance in an animal model of peritoneal metastasis from colon cancer

**DOI:** 10.1007/s10585-024-10283-5

**Published:** 2024-04-12

**Authors:** Vugar Yagublu, Bayram Bayramov, Christoph Reissfelder, Javahir Hajibabazade, Shalala Abdulrahimli, Michael Keese

**Affiliations:** 1 Department of Surgery, Medical Faculty Mannheim, Universitätsmedizin Mannheim, Heidelberg University, Theodor-Kutzer-Ufer 1-3, 68167 Mannheim, Germany; 2 Laboratory of Human Genetics, Genetic Resources Institute of Ministry of Science and Education, Baku, Azerbaijan; 3 Department of Natural Sciences, Western Caspian University, AZ1001 Baku, Azerbaijan; 4 Carver College of Medicine, University of Iowa, Bowen Science Building, 51 Newton Road, Iowa City, IA 52242-1009 USA; 5 Department of Vascular Surgery, Theresienkrankenhaus and St. Hedwigsklinik, Mannheim, Germany; 6 Medical Faculty Mannheim, DKFZ-Hector Cancer Institute, Heidelberg University, Mannheim, Germany

**Keywords:** Chemotherapy, Chemoresistance, Cell survival, Apoptosis, BMP7, Prss11

## Abstract

Chemotherapy drugs efficiently eradicate rapidly dividing differentiated cells by inducing cell death, but poorly target slowly dividing cells, including cancer stem cells and dormant cancer cells, in the later course of treatment. Prolonged exposure to chemotherapy results in a decrease in the proportion of apoptotic cells in the tumour mass. To investigate and characterize the molecular basis of this phenomenon, microarray-based expression analysis was performed to compare tHcred^2^-DEVD-EGFP-caspase 3-sensor transfected C-26 tumour cells that were harvested after engraftment into mice treated with or without 5-FU. Peritoneal metastasis was induced by intraperitoneal injection of C-26 cells, which were subsequently reisolated from omental metastatic tumours after the mice were sacrificed by the end of the 10th day after tumour injection. The purity of reisolated tHcred2-DEVD-EGFP-caspase 3-sensor-expressing C-26 cells was confirmed using FLIM, and total RNA was extracted for gene expression profiling. The validation of relative transcript levels was carried out via real-time semiquantitative RT‒PCR assays. Our results demonstrated that chemotherapy induced the differential expression of mediators of cancer cell dormancy and cell survival-related genes and downregulation of both intrinsic and extrinsic apoptotic signalling pathways. Despite the fact that some differentially expressed genes, such as BMP7 and Prss11, have not been thoroughly studied in the context of chemoresistance thus far, they might be potential candidates for future studies on overcoming drug resistance.

## Introduction

Colorectal cancer (CRC) is the third most commonly diagnosed cancer in men and the second most commonly diagnosed cancer in women, with over 1.8 million new cases and nearly 881,000 related deaths reported worldwide in 2018 [1]. Surgical removal of tumours and lymph nodes is considered the first-line treatment for early stage CRC [2, 3]. For patients affected by advanced-stage of CRC, chemotherapy is the most commonly used treatment option in adjuvant and palliative settings for achieving lasting remission and a definitive cure [4]. However, among CRC patients, those with advanced-stage CRC, are most likely to eventually develop resistance to both single and multiple chemotherapeutic agents despite initial positive responses [5].

Cancer cell resistance to chemotherapy is still a major problem. This results in the progression of local or metastatic disease, and metastasis is a leading cause of patient mortality from solid tumours [6]. Chemoresistance has been shown to be caused by numerous genes and multiple complex biological mechanisms, either intrinsic or acquired, including cancer stem cells (CSCs) and dormancy [7], decreased drug accumulation [8], reduced drug-target interactions [9], drug efflux mechanisms [5], alterations in drug targets and signalling transduction molecules [6], enhanced autophagy activity [10], epithelial–mesenchymal transition [11, 12], increased repair of drug-induced DNA damage, and apoptosis evasion [7, 13].

One of the key breakthroughs for advancing colon cancer treatment could be the overcoming drug resistance [5, 14]. Gaining in-depth knowledge on drug resistance mechanisms is of a paramount importance for developing a rational strategy to target resistant cancers [13, 15]. Although various studies have been conducted to clarify the molecular mechanisms of chemoresistance, the underlying mechanisms are still poorly understood. Therefore, to gain a better understanding of factors contributing to chemoresistance, novel targets should be identified.

We have previously reported the syngeneic mouse systems that express a FRET-based caspase-3 sensor that can be employed to analyse the therapeutic effectiveness of chemotherapy-induced apoptosis [16]. This syngeneic system allowed in vitro, in vivo, and ex vivo analysis of chemotherapy-induced apoptosis induction by optically monitoring the caspase-3 sensor state in the tumour cells. Tumour tissue analysis of 5-FU-treated mice revealed the selection of 5-FU-induced apoptosis-resistant tumour cells, which are referred to as cancer-repopulating cells (CRCs). These CRCs are known as CSCs and responsible for posttherapy relapse and metastatic colonization [17], which are the features most closely related to cancer-related death. This pilot study aimed to investigate and characterize the genetic basis of chemoresistance in chemotherapy-resistant cells. The expression analysis was performed by comparing tHcred2-DEVD-EGFP-transfected C-26 tumour cells that were harvested after engraftment into mice treated with or without 5-FU chemotherapy.

## Materials and methods

### Tumour cell culture maintenance

Wild-type C-26 murine colon carcinoma cells (referred to as C-26 cells), which can be used for construction of syngeneic models in BALB/c mice, obtained from the American Type Culture Collection™ (Manassas, VA). The cells were cultivated in DMEM medium supplemented with 10% (v/v) heat-inactivated foetal bovine serum, 100 U/ml penicillin, 100 lg/ml streptomycin, and 1% (v/v) glutamine in a humidified atmosphere of 95% air and 5% CO_2_ at 37 °C. Stock cultures were stored in liquid nitrogen and used for in vitro experiments within five passages. C-26 cells transfected with tHcred-DEVD-EGFP (referred to as C-26-c3s cells) were maintained under the same conditions as the wild-type cells. For in vivo studies, cells were harvested from subconfluent cultures. Cell viability was determined via trypan blue exclusion assays. The cell number was adjusted to 1 × 10^6^ cells in 0.5 ml of PBS for intraperitoneal injection to induce peritoneal metastases in mice.

### Animal experiments and reisolation of tumour cells

**Fig. 1:**
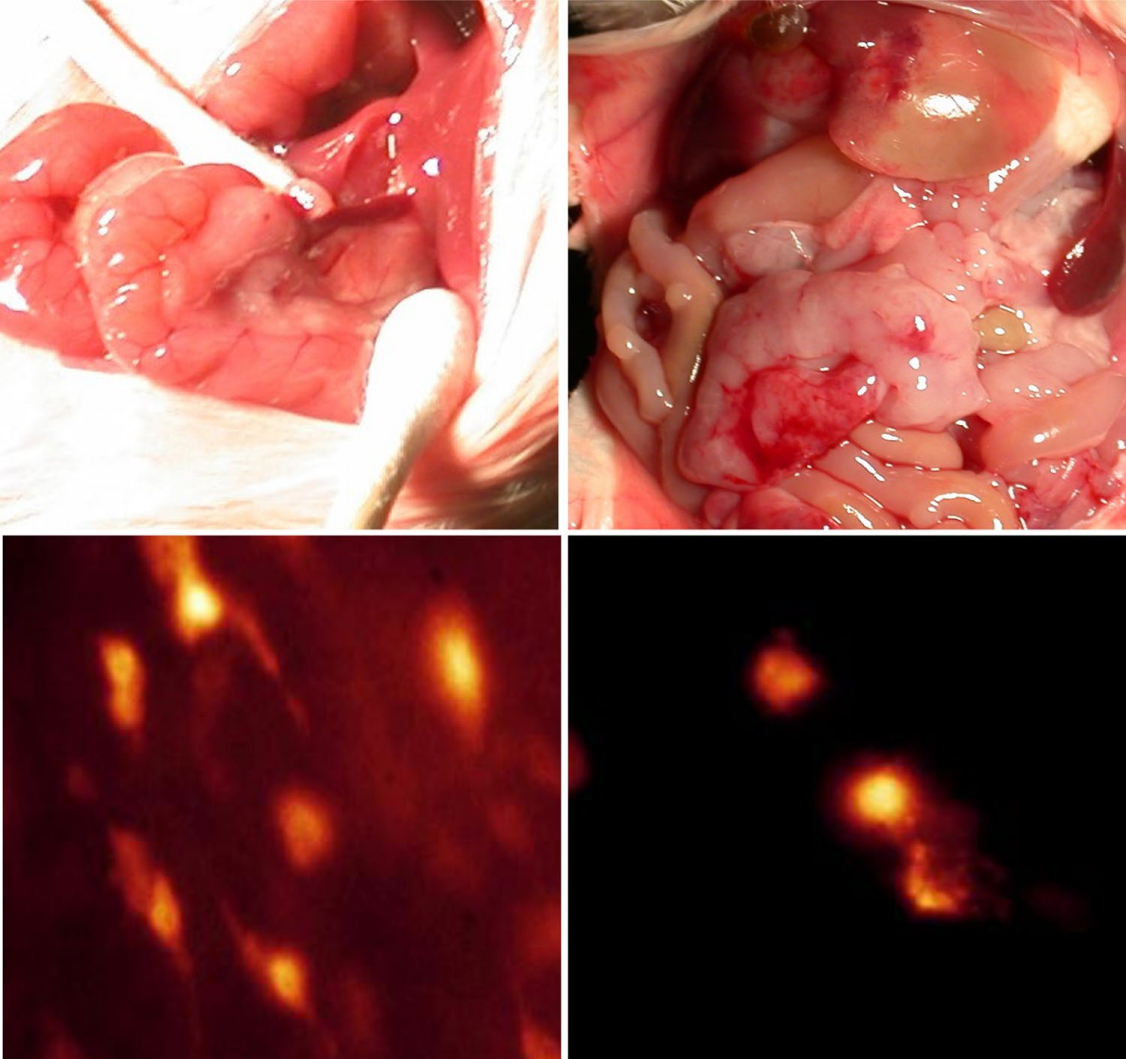
FLIM of caspase-3 sensor transfected C26 cells in metastatic omental tissue and following their reisolation. The top-left panel depicts a representative image of the normal peritoneal cavity in a mouse. The top-right panel presents an image of peritoneal metastasis generated by the injection of caspase-3 sensor-transfected C26 cells into the peritoneal cavity. The lower-left panel shows a FLIM image of metastatic omental tissue, while the lower-right panel shows a FLIM image after the C26 cells were reisolated and seeded onto a culture dish

The animal experiments were performed as described in our previous report [16]. Briefly, C26 cells carrying caspase-3 sensors were reisolated from omental metastatic tumours generated by intraperitoneal injection of the cells. C26 cells were reisolated from both omental tumor tissues of untreated controls and 5 days 5-FU-treated mice. We determined the sample size using the permutation method for sample size estimation in R software as previously described for small pilot datasets [18, 19]. The estimated range for group size was 4–6 mice per group. Each group, including both the control and treatment groups, consisted of five mice. To generate omental metastasis, the cells were injected (1 × 10^6^ cells in 0.5 ml of PBS) into the peritoneal cavity of BALB/c mice. The mice in the treatment group received 5-FU (30 mg/kg body mass) injection starting after the fifth day after tumour inoculation. The mice in both groups were sacrificed by the end of the 10th day after tumour injection. The omental tumour tissue specimens were carefully removed from the abdominal cavity. Directly after removal, the tissue samples were dissected into smaller blocks of 1 mm using a scalpel on the culture flask. The tissue pieces were incubated in an atmosphere of 100% humidity and 5% CO_2_ at 37 °C for 2–3 days in Dulbecco’s modified Eagle´s medium (DMEM). The cells that migrated away from the tissue pieces to the culture dishes, and the remaining necrotic tissue rests was removed during regular medium changes. FLIM Microscopy was used to confirm the purity of the C26 cells via detection of tHcred2-DEVD-EGFP-caspase 3-sensor. Figure 1 shows representative images from an animal experiment, that show the omental metastasis and FLIM features of C26 cells carrying caspase-3 sensor within omental tissue and after their isolation from the tissue and subsequent seeding onto a culture dish.

### Microarray and data analysis

For gene expression profiling, total RNA was purified (Qiagen RNeasy Micro Kit; Qiagen, Crawley, UK) from the reisolated C26 cells of 120 h-5-FU-treated and untreated mice (5 mice in each group; one mouse died during the experiment). Total RNA was used to generate cRNA, which was labelled with biotin according to methods recommended for CodeLink Expression Bioarray System (Amersham, UK). CRNA was then hybridized to DNA oligonucleotide probes attached to a gel matrix followed by secondary labelling and signal detection. The 25-mer microarrays (U133 Plus 2.0; Affymetrix, Santa Clara, CA) were used in our experiment. The microarrays were scanned with GenePix 4000B and analysed using GeneSpring 7.0 software.

### Validation of relative transcript levels with real-time semiquantitative RT-PCR

To validate the microarray data, semiquantitative real-time PCR was performed on an ABI Prism 7700 Sequence Detection System (PE Applied Biosystems, Foster City, CA, USA). cDNAs were synthesized using the same total RNA samples that were used for the microarray experiments. Total RNA samples (1 µg) were reverse-transcribed using the QuantiTect Reverse Transcription Kit with Integrated Genomic gDNA Wipeout Buffer (Qiagen) according to the manufacturer’s instructions. Primers for quantitative real-time PCR were purchased from Sigma/GenoSys (Steinheim, Germany). The sequences of primers used are listed in Table 1. Real-time PCR was performed with each specific primer pair using SYBR Green PCR Master Mix (PE Applied Biosystems). The data were analysed using the ABI Prism 7000 Sequence Detection System.

**Table 1:** List of primer sets used for quantitative real-time PCR

Gene	Forward primer	Reverse primers
Bid	5′ AATCATCCACAACATTGCCAGA-3′	5′-GCCTTGTCGTTCTCCATGTCT-3′
Dedd	5′-ACCGCATGTTCGACATCGT-3′	5′-CACGTCCATTTCGGATGAGTC-3′
Dap	5′-CAGTGTTCATCTCTGGCGTTAT-3′	5′-TTGGAGAAACGTGTTTGTCCAT-3′
Caspase-3	5′-TGGTGATGAAGGGGTCATTTATG-3	5′-TTCGGCTTTCCAGTCAGACTC-3′
Caspase-8	5′-TGCTTGGACTACATCCCACAC-3′	5′-TGCAGTCTAGGAAGTTGACCA-3′
Caspase-9	5′-TCCTGGTACATCGAGACCTTG-3′	5′-AAGTCCCTTTCGCAGAAACAG-3′
Notch1	5′-CCCTTGCTCTGCCTAACGC-3′	5′-GGAGTCCTGGCATCGTTGG-3′
Press11	5′-CGTATCGGAGGATGGACTGAT-3′	5′-TGGTCAATCTTGATAAGCGCAAT-3′
VEGFa	5′-CTTGTTCAGAGCGGAGAAAGC-3′	5′-ACATCTGCAAGTACGTTCGTT-3′
Ecsm1	5′-ACGAGTTTGGTATCTGCAAAGAC-3′	5′-GATGCTGAGTCACGCTCTGT-3′

## Results

**Fig. 2:**
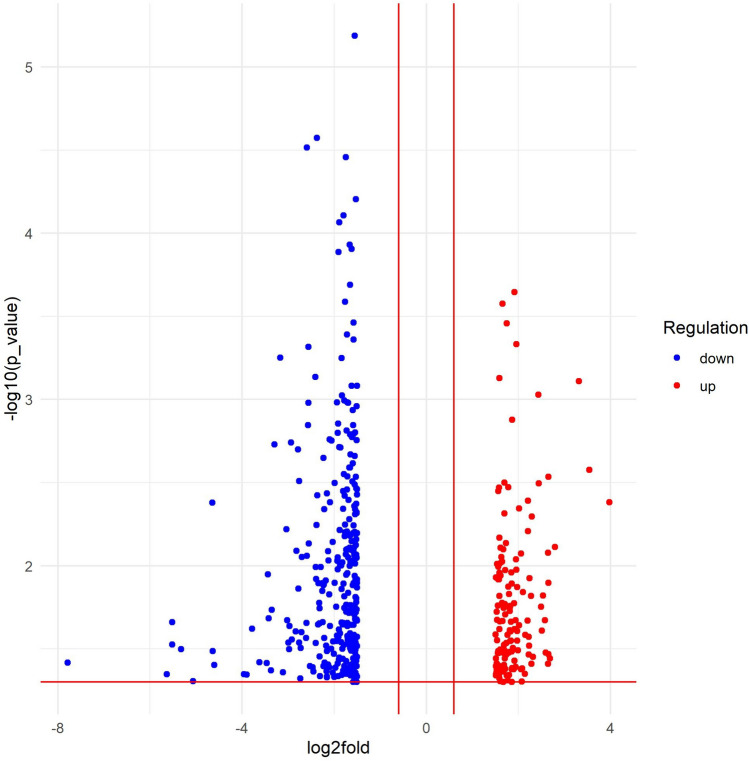
Comparison of mice teated with or without 5-FU for 120 h. The relative expression values for the two mouse experimental groups (mice treated with 5-FU for 120 h compared to untreated mice) are plotted, significantly differentially expressed genes are shown (*P* < 0.05)

Caspase-3-sensor-carrying C26 cells were used to generate peritoneal carcinomatosis models, followed by 5-FU treatment for five days (30 mg/kg body mass). The surviving cells subjected to this therapy were isolated for microarray analysis. Table 2 summarizes genes that are significantly differentially expressed in C26 tumour-bearing mice treated for 120 h with 5-FU in comparison with untreated mice, or that are associated with survival and apoptotic pathways. These genes could potentially contribute to 5-FU chemoresistance. Figure 2 schematically depicts the relative gene expression map for two experimental groups of mice—120 h 5-FU-treated mouse group compared to the untreated mouse group; where differentially expressed genes are shown.

**Table 2:** List of all genes of interest from an Affymetrix whole-genome gene expression microarray analysis of RNA from 120 h treatment of mice (*n* = 4) bearing C26 tumour cells treated with 5-FU for 120 h compared with untreated mice (*n* = 5)

GeneID	Gene name	Fold change	P	Function
Significantly upregulated genes				
56213	Prss11, Protease, serine, 11(IgG binding)	3.018	0.000591	Regulation of cell growth
22339	VEGF A, Vascular endothelial growth factor A	1.528	0.0136	Regulation of angiogenesis
71690	Esm1, Endothelial cell-specific molecule 1	2.33	0.00168	Regulation of cell growth
54123	Irf7, Interferon regulatory factor 7	2.12	0.0467	Regulation of transcription
69583	Tnfsf13, Tumor necrosis factor (ligand) superfamily, member 13	1.301	0.0104	Immune response and positive regulation of cell proliferation
50930	Tnfsf14, Tumor necrosis factor (ligand) superfamily, member 14	1.042	0.0158	Immune response
14455	Gas5, Growth arrest specific 5	1.117	0.0382	Growth arrest
21812	Tgfbr1, Transforming growth factor, beta receptor I	2.163	0.0003	Negative regulation of Apoptosis
260299	Igsf4c, Immunoglobulin superfamily, member 4 C	2.843	0.00576	Negative regulation of VEGF signaling pathway
108075	Ltbp4, Latent transforming growth factor beta binding protein 4	2.141	0.00359	Transforming growth factor beta binding
68024	Hist1h2bc, Histone 1, H2bc	1.906	0.0293	Chromosome organization and biogenesis
19090	Prkdc, Protein kinase, DNA activated, catalytic polypeptide	3.1	0.0129	DNA repair
237858	Tusc5, Tumor suppressor candidate 5	3.027	0.00695	Endosome to plasma membrane protein transport
14127	Fcer1g, Fc receptor, IgE, high affinity I, gamma polypeptide	2.145	0.0412	Positive regulation of tumor necrosis factor-alpha biosynthesis
74191	P2ry13, purinergic receptor P2Y, G-protein coupled 13	2.365	0.044	G protein-coupled receptor signaling pathway
12162	Bmp7, Bone morphogenetic protein 7	2.993	6.34 × 10^−5^	Cytokine activity; growth factor activity;
230316	Egfl5, EGF-like-domain, multiple 5	1.482	0.0381	Structural molecule activity
Significantly downregulated genes				
12122	Bid. BH3 interacting domain death agoinst	0.586	0.0294	Regulation of apoptosis
21945	Dedd, Death effector domain-containing	0.714	0.0278	Regulation of apoptosis
223453	Dap, Death-associated protein	0.72	0.00323	Induction of apoptosis
12369	Caspase 7	0.594	0.00236	Regulation of apoptosis
18128	Notch1, Notch gene homolog 1 (Drosofila)	0.678	0.0432	Regulation of transcription
12018	Bak1, BCL2-antagonist/killer 1	0.654	0.0183	Caspase activation via Cytochrome c
12043	Bcl2, B-cell leukemia/lymphoma 2	0.712	0.0202	Anti-apoptosis
68083	Pak1ip1, PAK1 interacting protein 1	0.73	0.00175	Negatively regulates the PAK1 kinase
16451	Jak1, Janus kinase 1	0.684	0.000581	Intracellular signaling cascade
268287	Akap7, A kinase (PRKA) anchor protein 7	0.776	0.00377	Transmembrane receptor protein serine/threonine kinase signaling pathway
11637	Ak2, Adenylate kinase 2	0.708	0.00782	Adenylate kinase activity, ATP binding, kinase activity
21928	Tnfaip2, Tumor necrosis factor, alpha-induced protein 2	0.797	0.0298	Angiogenesis
56745	C1qtnf1, C1q and tumor necrosis factor related protein 1	0.763	0.0403	Receptor activity
14457	Gas7, Growth arrest specific 7	0.501	0.00657	Development and neurogenesis
15516	Hspcb, Heat shock protein 1, beta	0.728	0.0472	Protein folding, response to heat
66667	Hspbap1, Hspb associated protein 1	0.678	0.0286	2-oxoglutarate-dependent dioxygenase activity
15528	Hspe1, Heat shock protein 1 (chaperonin 10)	0.807	0.0172	Unfolded protein binding
21814	Tgfbr3, Transforming growth factor, beta receptor III	0.408	0.0226	Angiogenesis, negative regulation of Apoptosis
16193	Il6, Interleukin 6	0.441	0.022	Immune response; programmed cell death
54725	Igsf4a, Immunoglobulin superfamily, member 4 A	0.429	0.046	
57269	Olfr1507, Olfactory receptor 1507	0.446	0.00927	G-protein coupled receptor activity
14084	Faf1, Fas-associated factor 1	0.475	0.0335	Potentiates Fas-induced cell killing
11658	Alcam, Activated leukocyte cell adhesion molecule	0.422	0.000345	Cell adhesion; signal transduction
26411	Map4k1, Mitogen activated protein kinase kinase kinase kinase 1	0.478	0.00204	ATP binding; kinase activity; protein kinase activity
17311	Kitl, Kit ligand	0.488	0.00342	Growth factor activity; protein binding; stem cell factor receptor binding
15903	Idb3, Inhibitor of DNA binding 3	0.5	0.0291	Protein binding; protein domain specific binding
Genes with no statistically significant regulation				
12367	Caspase 3	0.855	*P* > 0.05	Induction of apoptosis
12370	Caspase 8	0.804	*P* > 0.05	Induction of apoptosis
12371	Caspase 9	0.868	*P* > 0.05	Induction of apoptosis
14102	Tnfrsf6 (aka Fas). Tumor necrosis factor receptor superfamily, member 6	0.471	*P* > 0.05	Apoptosis and immune response

To validate the results of the microarray analysis, several representative genes were analysed using semiquantitative real-time PCR (Table 3). These genes were Bid, Dedd, Dap, Caspase 3, Caspase 8, Caspase 9, Notch1, Prss11, VEGF-A, and Ecm1. Consistent with the microarray data, similar regulatory patterns of these genes were detected.

**Table 3:** Microarray outcomes were validated using quantitative real-time PCR with representative genes. “-“ indicates downregulation, “+” indicates upregulation, “*” indicates significant differential expression

Gene names	Microarray results	Semi-quantitative real-time PCR
Bid, BH3 interacting domain death agonist	− 1.71*	− 2.391*
Dedd, death effector domain-containing	− 1.224*	− 1.4*
Dap, death-associated protein	− 1.38*	− 1.312*
Caspase 3	− 1.116	− 1.296
Caspase 8	+ 1.24	+ 1.006
Caspase 9	− 1.15	− 1.1
Notch1, notch gene homolog 1	− 1.47*	− 1.45*
Prss11, protease, serine, 11(IgG binding)	+ 3.018*	+ 3.907*
VEGF A, vascular endothelial growth factor A	+ 1.526*	+ 2.158*
Esm1, endothelial cell-specific molecule 1	+ 2.33*	+ 2.92*

Using the DAVID Bioinformatics Database, we defined the specific pathways involving the 47 RNA molecules of interest, that contribute to the onset and progression of cancer cell survival and chemoresistance.

In addition to the data collected and processed from various scientific research articles, we decided to include a discussion of our findings from the DAVID Bioinformatics Database, through which we analysed the list of genes that were differentially expressed in cancer cells that survived chemotherapy according to the identifier ENTEREZ_GENE_ID. We used the “Functional Annotation Report” of this program with the primary goal of determining whether the genes overexpressed in mice treated with chemotherapy participate in numerous common pathways leading to cancer cell survival and apoptosis suppression. Among the 47 identified RNAs, 15 were found to be involved in various cancer pathways simultaneously. A total of 14 gene products (Fas, Notch1, Bak1, Bid, Htra1, Bmp7, Casp3, Casp7, Casp8, Casp9, Idb3, Il6, Prkdc, and Tgfbr1) are involved in the positive regulation of apoptosis, with 5 of them (Fas, Bid, Casp3, Casp8, Casp9) and Bcl2 are members of the p53 signalling pathway; 9 of gene products participate in negative regulation of apoptosis (Fas, Bcl2, Casp3, Hsp90ab1, Kitl, Il6, Prkdc, Tgbr1, and Vegfa). We also detected the involvement of 7/47 analysed genes (Bcl2, Jak1, Casp9, Hsp90ab1, Il6, Kitl, and Vegfa) in the PI3K–Akt pathway.

## Discussion

It is well known that chemotherapy drugs efficiently eradicate rapidly dividing differentiated cells by inducing cell death at earlier stages but poorly target slowly dividing cells, including CSCs and dormant cancer cells [20]. Our previously published work demonstrated this phenomenon in a mouse model of peritoneal carcinomatosis of colon carcinoma, where under longer chemotherapy exposure, a lower proportion of apoptotic cells in the tumour mass was detected [16]. To gain a better understanding of the mechanisms involving reversible genetic alterations that could lead to chemoresistance, we analysed of genes of interest in 5-FU-treated mice under chemotherapy treatment to determine their relation to chemoresistance development by determining whether the corresponding gene products promote the survival of cancer cells. The results revealed the downregulation of both intrinsic and extrinsic apoptotic signalling pathways and the upregulation of some mediators of cancer cell dormancy and survival-related genes. The critical mediators of intrinsic and extrinsic apoptotic signaling pathways, such as Bak1, Casp7, Bid, Bcl2, was significantly downregulated in cancer cells exposed to 5-FU. Moreover, some of the important genes of these pathways, such as Casp9 and Casp3, were not regulated.

One of the most likely mechanisms underlying the acquired resistance to 5-FU-induced apoptosis is the differential expression of Fas pathway members. Several genes in this pathway, such as Bid, DEDD, Caspase 7, and DAP were significantly downregulated. Fas (also known as Tnfrsf6) is a death domain-containing member of the TNF receptor superfamily that plays an important role in regulating apoptosis, the pathogenesis of several malignancies, and immune system disorders [21]. Crosslinking of the Fas receptor through Fas ligands or agonistic antibodies results in the formation of death-inducing signal complexes, which include the adaptor proteins FADD/MORT-1 and Caspase 8 [22]. Interestingly, the Caspase 8 and the Tnfrsf6 gene, which are essential for apoptosis execution, were not differentially expressed in C26 cells that survived the chemotherapy. Cleavage of BID (Bcl-2 family proapoptotic protein required for death receptor-mediated apoptosis) by Caspase 8 induces its strong proapoptotic activity, which eventually causes mitochondrial damage and, in due course, cell shrinkage and nuclear condensation [23]. DEDD (DEFT), known as a death effector domain-containing protein, accelerates Fas-induced apoptosis by interacting with FAS-associated death domain-containing protein (FADD) and caspase-8 [24, 25]. Another differentially expressed proapoptotic factor, DAP, is still being studied in terms of its functional role in pathways leading to apoptosis [26]. DAP is a negative regulator of autophagy; that is, it can prevent or suppress authophagy [27]. DAP-kinase was discovered to have potent tumour-suppressive properties, linking the control of apoptosis to metastasis [28]. Caspase 7, which was also downregulated in our study, has been demonstrated to be activated during Fas-induced apoptosis [29]. The downregulation of the aforementioned members of the Fas pathway is consistent with the fact that this pathway is known to promote apoptosis; therefore, its downregulation might contribute to the progression of chemotherapy resistance through apoptosis evasion. However,there is a preliminary study that seems to be contradictory; the authors proposed that Fas signalling induces epithelial–mesenchymal transition (EMT), which promotes motility and metastasis, in gastrointestinal cancers [30]. Efforts are being made to develop cancer therapies based on Fas signalling, but these agents need to be administered cautiously because activation of this pathway can not only induce apoptosis, but also induce resistance to chemotherapy [31]. This finding is of significance importance in our research because it might explain why, as mentioned earlier, Fas (Tnfrsf6) is involved in both the negative and positive regulation of apoptosis according to functional clustering based on the DAVID Bioinformatics Database; hence, more comprehensive studies are needed to clarify the role of the Fas pathway, and eventually advance chemotherapy-based treatments.

So-called survival signalling pathways counter apoptosis signalling pathways. In this context, several genes in the MAPK1/MAPK3 signalling pathway (Kit, Il6, Jak1, and Kitl) were significantly downregulated after 5-FU treatment. Because of its intrinsic complexity and diverse crosstalk with other signalling pathways, the regulation of this pathway remains unclear, as does its involvement in chemoresistance [32].

We also showed the upregulation of several genes in PI3K-Akt pathway (Bcl2, Jak1, Casp9, Hsp90ab1, Il6, Kitl, VEGF A), which is an intracellular signal transduction pathway, a so-called cell survival pathway, that promotes proliferation, cell survival, metabolism, growth, and angiogenesis in response to extracellular inputs [33, 34]. A wide range of human cancers, including breast, colon, gastric, lung, and prostate cancers have been shown to be associated with PI3K activity [35, 36]. Further evidence has shown that Akt (protein kinase B), a downstream kinase of PI3K, is also involved in malignant transformation [37]. Inhibition of the PI3K-Akt pathway could be an advantageous strategy for developing state-of-the-art chemotherapeutic treatment methods and is currently being intensively investigated as a potential cancer treatment strategy [36, 38].

*BMP7* gene (bone morphogenetic protein-7), which belongs to the transforming growth factor-β superfamily and is associated with the dormancy of cancer cells, including CSCs, was significantly upregulated in tumour cells treated with 5-FU in a manner dependent on the activation of p21, p38 MAPK, and N-myc downstream-regulated gene 1 via BMP receptor-2 [39]. BMP7 can positively regulate EMT, a process that increases the rate, frequency, or extent of epithelial-to-mesenchymal transition and negatively regulates cell death [40]. EMT, in turn, plays a pivotal role in predicting cancer cell growth into macrometastases [12]. Verschi et al. showed that BMP7 is highly expressed in low-grade CRC patients with both colon adenoma and adenocarcinoma, suggesting that this phenomenon is an early event in CRC [41]. This group demonstrated that BMP7 exerts potent antitumour activity by inducing the differentiation of PIK3CA wild-type CRC stem cells (wt CR-CSCs) and suggested that BMP7-based combination therapies may represent potential novel treatment options for CRC.

Additionally, vascular endothelial growth factor (VEGF-A) and Notch1, which are both key factors in angiogenesis, were considerably upregulated in chemotherapy-treated cells. As chemotherapy augments nutrient and oxygen dependency [42], it can be presumed that tumour cells could benefit from the promotion of angiogenesis. VEGF-A is a critical stimulator of angiogenesis because its binding to VEGF receptors stimulates endothelial cell migration and proliferation, both of which serve as keys in the development of new blood vessels [43]. Furthermore, VEGF-A controls vessel sprouting and branching by inducing the expansion of endothelial tip cells, and increasing vascular permeability, which, in turn, might also contribute to angiogenesis and tumour progression [44]. ESM-1 overexpression could be caused by an analogous mechanism. Hhex-mediated suppression of ESM-1 is required for normal vascular endothelial function, tumour vasculogenesis, and cancer progression [45]. In this context, Kang et al. suggested that ESM-1 may be a useful therapeutic target for CRC [46].

Notch1 is also a negative regulator of cell death, and ligands of Notch1 play an important roles in cell fate determination [47]. Although the expression of Notch1 and its ligand in the vascular endothelium and defects in the vascular phenotypes of targeted mutants in the Notch pathway have already been described [48], the specific signalling pathways controlling their expression remain unknown [49].

The HtrA1 gene was significantly upregulated in chemotherapy-selected cells. HtrA1 has already been hypothesized to function as a tumour suppressor [50]. The first clinical study on melanoma was carried out by Baldi and colleagues [51], who reported significant HtrA1 upregulation in primary tumours compared with that in metastases, and suggested that HtrA1 expression could be an indicator of disease progression. Downregulation of HtrA1 protein is associated with poor survival in mesothelioma [52], hepatocellular carcinoma [53], and breast cancer [54]; in the latter study, nodepositivity was associated with shorter survival. HtrA1 downregulation has also been connected to a poor chemotherapy response in patients with gastric cancer [55]. These findings suggest a possible prognostic role for HtrA1 expression.

## Conclusion

Based on our results and discussion, it can be concluded that understanding the exact mechanism of chemoresistance is crucial for overcoming this challenge. An auspicious and powerful approach for the treatment of resistant and recurrent neoplastic diseases could be provided by the reprogramming tumour cells to undergo drug-induced apoptosis by means of novel targeted agents. This can be achieved by downregulating the involved dysregulated antiapoptotic factors or activation of proapoptotic factors in tumor cells. Deeper research works on the intrinsic cell kinetics and mechanisms that promote and sustai cancer cells in a dormant state and the long-term consequences of dormancy, are critical for improving current therapeutic treatment outcomes.
